# Improving Surgical Access in Rural Africa through a Surgical Camp Model

**DOI:** 10.1155/2016/9021945

**Published:** 2016-06-16

**Authors:** M. Galukande, O. Kituuka, E. Elobu, J. Jombwe, J. Sekabira, Elissa Butler, J. Faulal

**Affiliations:** ^1^Department of Surgery, College of Health Sciences, Makerere University, Kampala, Uganda; ^2^Department of Surgery, Mulago National Referral Hospital, Kampala, Uganda; ^3^University of Minnesota Medical School, Minneapolis, MN 55455, USA

## Abstract

*Introduction*. Surgical camps are preplanned activities where volunteer surgical teams congregate at specified place(s) and perform a wide range of mostly elective procedures for a limited period of time. This is usually at no cost to the patients, who belong to vulnerable (poor and hard to reach) communities. We describe a surgical camp model and its challenges as a means of improving access to surgical services.* Methods*. A cross-sectional descriptive study. Data from a recent Association of Surgeons of Uganda surgical camp were collected and analyzed for demographics, costs, procedure types, and rates and, in addition, challenges encountered and solutions. Personnel that participated in this exercise included specialist surgeons, surgical residents, medical officers, clinical officers, anesthetists, and theater nurses (a total of 121 staff).* Results*. In total, 551 procedures were performed during a four-day-long camp. Mean age was 35 years (SD 23), M : F ratio was 2 : 1. Herniorrhaphy, skin lump excision, hydrocelectomy, and thyroidectomy formed 81% of all the procedures. Average cost per procedure was $73 USD.* Conclusion*. Surgical camps offer increased access to surgical services to vulnerable populations. Hernias and goiters were most common. Surgical camps should become an integral part of the Health Service delivery in low-resourced environments.

## 1. Introduction 

Surgical conditions are neglected in healthcare systems in Sub-Saharan Africa [1, 2]. There is limited access to surgical services and accordingly low surgical output compared to richer nations [3, 4]. The reasons for limited access to surgical services are numerous, including but not limited to inadequate Human Resources for Health (HRH), limited surgical supplies, and lack of transportation to get to the distant health facilities that can offer the appropriate surgical services [5, 6].

In response to these realities, the Association of Surgeons of Uganda (ASOU) piloted the first surgical camp in 2001 in northern Uganda, a 6-hour drive from the capital city of Kampala. Due to the success of this first camp, general surgeons continue to offer annual camps. Additionally, subspecialty camps occur regularly, such as for open heart surgery and plastic-cleft lip repairs [7, 8]. On average, 1 to 2 surgical ASOU endorsed camps are carried out every year, in different regions of the country each time.

Surgical camps are preplanned activities were volunteer surgical teams congregate at a specified place and perform a wide range of mostly elective procedures for a limited and specified period of time normally a week or little more, usually at no cost to the patient. Several fundraising activities precede the camp to cover direct costs, such as transportation, food, and water at the venues. The sources of funds are from the business private sector, individuals, and Ministry of Health. There is normally no monetary compensation for the personnel: services are offered pro bono.

The purpose of this study therefore was to describe this surgical camp model as an approach to improving access to surgical services for vulnerable communities and estimate the unmet burden of surgical disease in the areas where the camps took place.

## 2. Materials and Methods

### 2.1. Study Design

This is a cross-sectional, descriptive study using both quantitative and qualitative data collection methods.

### 2.2. Study Settings

The study took place in Northeastern Uganda at 8 rural sites, including 3 district hospitals, 4 health center level IVs, and 1 regional hospital, in the month of July 2013.

All sites had operating rooms, postoperative recovery areas, and the admission space for those who required it. The teams at the sites included specialist general surgeons, nurses, medical officers, and medical students. Patient follow-up was conducted by the resident health worker at the host sites following routine standard protocols.

### 2.3. Data Collection

Before the camp, mobilization activities took place to recruit patients, including but not limited to radio announcements, announcements at special gatherings such as church services, advertisements at health facilities, and house-to-house visits by the village health teams and the local council committee.

A day or two prior to commencement of surgeries, clinical screening was conducted, and those eligible for surgery were selected. Clinician's judgment, based on history and physical exam, determined fitness for surgery and anesthesia, as laboratory and imaging capacity was limited. Personnel that participated in the camp included 20 specialist surgeons, 6 surgical residents, 6 medical officers, 7 clinical officers, 17 anesthetists, and 55 theater nurses (a total of 121 staff in all).

Operating logs were filled during the camp, which included the date of procedure, patient age and gender, clinical diagnosis, operation performed, and type of anesthesia. Data were extracted from the operating logs. Camp summary reports were submitted, which included challenges encountered and solutions undertaken. Direct expenditure details were obtained from the finance secretary of the Association/camp.

### 2.4. Data Analysis

Data were entered in Excel from paper operating logs and analyzed. Variables considered were age, gender, diagnosis, procedure done, direct cost per patient, and type of anesthesia. Data from the summary reports and interviews were collected into themes and presented in a table.

### 2.5. Ethical Issues

All patients gave their informed written consent for the procedures done.

## 3. Results 

### 3.1. Patient Demographics

**Figure 1:**
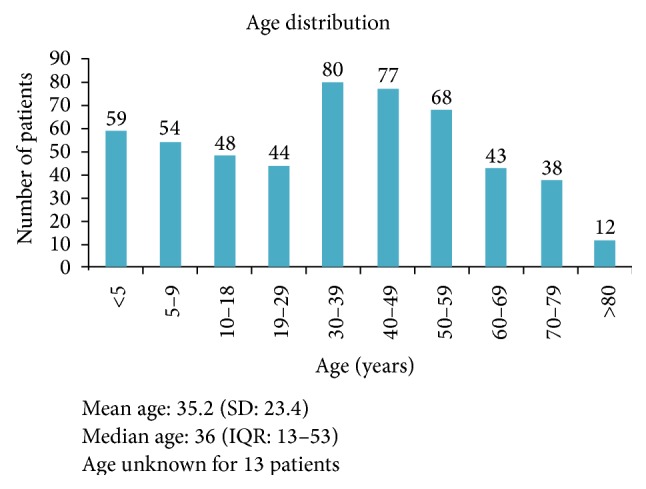
Age distribution of patients receiving surgery at the surgical camp, Uganda, 2013.

A total of 536 patients were operated on and 551 procedures were performed over a period of 4 days across 8 sites. Mean age was 35 years (SD 23) and gender ratio was 2 : 1 (male : female). The age distribution had a bimodal trend as shown in Figure 1. However, for hernia repair and hydrocelectomy, the age distribution was constant (see Table 3).

### 3.2. Clinical Diagnoses and Surgical Procedures

Of the 551 procedures, the most common diagnoses were inguinal hernia, hydrocele, lipoma, epidermoid cyst, and goiter contributing to 70% of all diagnoses (see Table 1). The four most common procedures were herniorrhaphy, skin lump excisions, hydrocelectomy, and thyroidectomy, accounting for 81% of all procedures (see Table 2). Immediate postoperative mortality was zero (1–4 days). In total, 70% were done under local or spinal anesthesia.

### 3.3. Challenges

Several logistical challenges were encountered, including power outages, inadequate sterilization and anesthetic equipment, lack of running water, and inadequate surgical sets. Others included an overwhelming number of patients, inadequate nursing personnel to cover the recovery bays, and limited laboratory and imaging investigative capability (see Table 4).

### 3.4. Expenses

The gross expenditure on direct costs was 100,000,000 Uganda Shillings ($40,000). This converts to 187,000 Uganda Shillings (or $75) per patient and 182,000 Uganda Shillings (or $73) per procedure.

### 3.5. Types of Anesthesia Used at the Surgical Camp in Uganda

There were three types of anesthesia as follows:

 General anesthesia 109 (30%).
 Locoregional anesthesia 206 (57%).
 Spinal anesthesia 97 (13%).

## 4. Discussion 

The surgical camp model for providing surgical services to vulnerable or underserved populations in Africa is not new, and in the case of Uganda and ASOU this has been done for the past decade [7]. However, data on patient demographics and the scope of surgical procedures have not been previously documented in the literature.

Persons with surgical disease are young and form the bulk of the Ugandan productive work force. The bimodal distribution of age showed that the paediatric (below 18 years) population and those between 30 and 60 years represent the bulk of the surgical burden of disease. This emphasizes the need for paediatric surgical skills in surgical training for medical officers.

Males were overrepresented, likely due to the high burden of inguinal hernias and hydroceles. Although the procedure per population rate was estimated at 12.5 : 100,000 in this review, at some sites it was as high as 146 : 100,000, close to what was estimated by Nordberg [9] in 1984, three decades ago. Inguinal hernia and hydrocele occurrence were in equal proportions throughout age distribution.

The most common conditions seen in our study were hernia, hydrocele, and goiter. These procedures are within the realm of competence of a medical officer (nonspecialist physician) if properly trained and supported [10]. What is also true is that surgical camps environment is a platform for apprenticeship: the less experienced surgeons or trainees work with more experienced colleagues to learn more in a practical setting handling high volumes of surgical cases in a short period of time.

Locoregional anesthesia was used in 70% of cases. This aligns with recommendations made previously on the basis of cost of care and safety.

This study also allowed us to estimate procedure per population rate for the less common surgical conditions. The prevalence of correctable surgical congenital anomalies in Uganda is not known. In total, 4% of all procedures done were for congenital anomalies. Gluteal fibrosis or gluteal muscle contracture caused by repeated intramuscular quinine injections accounted for 3% of surgical diagnoses. It accounted for 3% of surgical diagnoses. GMC, first reported by Valderrama, is a clinical syndrome pathologically characterized by degeneration, necrosis, and fibrosis of the gluteal muscles and fascia, leading to serious limitation of hip movements [11, 12]. Malaria is endemic in Uganda, and in some places injectable quinine is used indiscriminately.

Several challenges were encountered; screening for comorbidities such as NIDDM (non-insulin dependent diabetes mellitus) and cardiorespiratory diseases was limited to clinical assessment without lab and imaging for most patients, yet we know there is a significant burden of prediabetic and diabetic states among Ugandans [11]. The consequences of not screening are not known in this context.

Whereas these populations have close geographical access to health centers, they likely delay in access to appropriate care due to a number of reasons including lack of skilled manpower at the health centers, drug stock-outs for anesthetics, and health centers prioritizing resources for emergency procedures like Cesarean sections [13]. During the camp, several logistical challenges were faced ranging from power outages, inadequate sterilization capacity, limited surgical instruments, limited operating room space and intermittent supply of running water. These are not new [2, 5, 14]; they impede access to surgical services even for stop-gap approaches like surgical camps. Documenting these challenges is part of advocacy to mobilize resources and engage those that have the power to prioritize available resources for service.

What has also been highlighted here (albeit crudely) is the cost of surgical intervention. The cost of surgical intervention was $72.50 per procedure. This cost however was further subsidized by the infrastructure that already exists.

What is clear from this study is that the scope of conditions that were seen over ten years ago is still the same. However, a population-based estimate of the burden of surgical disease [3] and regular documentation of the camps' outcomes have not been done. Estimates indicate that surgery can address 7% of DALYs (disability-adjusted life years) that occur in Africa [15] and this burden is most probably increasing rapidly [14].

Organizing a camp successfully requires a clear objective, a group of individuals or organization(s), finance(s), and an appropriate target population (beneficiary group). In addition, there was an agreed work plan with the involvement of all stakeholders including local leaders, hosting health centers, sponsors, Ministries of Health, and the personnel that carry out the procedures. Publicity of these activities is encouraged in order to promote the service, gain support, and ensure sustainability on the long term.

## 5. Study Limitations 

The data for screened patients that did not qualify for surgery were unavailable. The catchment population is an estimate; there may be overlap of health facility catchment areas, and previously done procedures in the area/catchment were not known. No postoperation data was collected to assess postoperative complications which may inform future camps.

## 6. Conclusion 

Surgical camps improve access of surgical services to vulnerable populations. Inguinal hernias formed the bulk surgical disease encountered. Hydroceles and thyroid disease also contribute significantly to the burden of disease in Sub-Saharan Africa. Surgical camps should become an integral part of health service delivery in rural Africa.

## Figures and Tables

**Table 1:** Surgical procedures at a campsite in Uganda, 2013.

	Amuria^†^	Kaberamaido^†^	Soroti^▲^	Ngora^*∗*^	Katakwi^†^	Kumi^*∗*^	Aturur^*∗*^	Serere^†^	*Total*
Herniorrhaphy	13	26	20	26	13	4	21	55	*178*
Skin excisions	12	10	16	12	18	8	26	29	*131*
Hydrocelectomy	26	19	6	6	24	3	11	10	*105*
Thyroidectomy	0	0	15	10	0	9	0	1	*35*
Gluteal fibrosis release	0	0	0	0	0	17	1	0	*18*
Anorectal procedures^1^	0	2	7	1	1	0	6	0	*17*
Wound-related procedure^2^	2	1	7	0	1	0	4	1	*16*
Laparotomy	0	0	3	0	0	4	1	2	*10*
Obstetric^3^ conditions	6	0	0	0	0	0	1	1	*8*
Hysterectomy	0	0	0	1	0	0	5	1	*7*
Orchiopexy	0	5	0	0	0	0	1	1	*7*
Orthopedic procedures	0	1	3	0	1	0	0	0	*5*
Appendectomy	0	0	1	0	0	1	0	2	*4*
Other procedures	0	0	2	5	0	1	2	0	*10*

*Total*	*59*	*64*	*80*	*61*	*58*	*47*	*79*	*103*	*551*

^1^Hemorrhoidectomy, parasagittal anorectoplasty, lateral internal sphincterotomy, and manual anal dilation.

^2^Incision and drainage, debridement, and surgical toilet.

^3^Cesarian section and uterine evacuation.

^*∗*^District hospitals with a 100-bed capacity, all state owned public hospitals.

^†^Health center IVs, outpatient facilities with 20-bed in-patient facilities.

^▲^A regional referral hospital, 250 beds with some specialist services.

**Table 2:** Showing diagnosis by age, campsite in Uganda, 2013.

Procedure	Age groups					Unknown age	Total
	<5	5–18	19–39	40–59	≥60		
Inguinal hernia	29	29	23	29	27	6	143
Hydrocele	10	19	12	36	33	2	112
Lipoma	2	7	14	19	14	1	57
Goiter	0	0	16	15	3	1	35
Epidermoid cyst	2	5	11	8	5	1	32
Other hernias	0	1	8	9	8	1	27
Congenital conditions	13	5	2	1	0	0	21
Gluteal fibrosis	0	18	0	0	0	0	18
Anorectal	0	1	10	5	1	0	17
Infection	1	4	3	6	2	0	16
Tumor	2	3	6	3	1	1	16
Gynecologic	0	1	6	6	0	0	13
Intra-abdominal masses	0	3	3	4	1	0	11
Ganglion cyst	0	2	2	1	2	0	7
Obstructed labor	0	2	4	0	0	0	6
Trauma	0	1	3	2	0	0	6
Other procedures	0	6	4	3	1	0	14

Total	59	107	127	147	98	13	551

**Table 3:** Procedure per population rates, campsite in Uganda, 2013.

Site	Catchment population (2010/11 estimates)	Category and number of procedures		Procedure: per 100,000 of population
		Procedure	Number	
Amuria	315,900	Hernia	13	1 : 4.1
		Hydrocele	26	1 : 8.2
		Goiter	0	—

Kaberamaido	195,400	Hernia	18	1 : 9.2
		Hydrocele	25	1 : 12.8
		Goiter	0	—

Katakwi	153,600	Hernia	10	1 : 6.5
		Hydrocele	24	1 : 15.6
		Goiter	8	1 : 5.2

Serere	176,500^*∗*^	Hernia	45	1 : 25.5
		Hydrocele	10	1 : 5.7
		Goiter	13	1 : 7.4

Ngora	101,900^*∗*^	Hernia	18	1 : 17.7
		Hydrocele	6	1 : 5.9
		Goiter	10	1 : 9.8

Kumi and Aturur	13,000	Hernia	19	1 : 146
		Hydrocele	15	1 : 115
		Goiter	9	1 : 69

Soroti	241,200	Hernia	20	1 : 8.3
		Hydrocele	6	1 : 2.5
		Goiter	15	1 : 9.9

Overall	1,197,500	Hernia	147	1 : 12.5
		Hydrocele	112	1 : 10
		Goiter	35	1 : 2.9

^*∗*^2002 population census estimates.

**Table 4:** Challenges reported, solutions, and future plans.

Domain	Challenges	Solutions
Water and electricity	Lack of running water at some sites Prolonged power outages at two sites	Patients and/or their attendants to provide 20 litres of water each Using a generator (consider standby generators in future surgical camps)
	Inadequate number of anesthetists No anesthetic machine	Predetermining personnel needs and secure personnel (anesthetists) beforehand

Sterilization and supplies	Limited capacity to sterilize (due to inadequate number of autoclaves), power outages, and inadequate linen supplies Drugs and surgical sundries were in short supply	Better projections and resource mobilization for future camps Considering hiring autoclaves Contact nearby hospitals to participate and share

Equipment and instruments	Equipment and instruments were limited (surgical sets, anesthesia equipment) There was a concurrent ophthalmology camp going on We had only one oxygen source	Doing better projections, hiring equipment and instruments Considering portable oxygen supply

Human resource	Several patients with gynecological conditions came yet we had no gynecologists Operating theater condition: some were very old, dilapidated Few staff in the theater to help in coordination and patient flow Inadequate postoperative nursing manpower One of the team members fell sick	Including gynecologists in future camps Instituting quality assurance and safety guidelines and agreeing on the minimum standards Getting required personnel to commit before the camp begins

Demand for service	Overwhelming number of cases Some pediatric cases could not be worked on	Planning triage days before the camp begins and generating manageable operating lists

Technical operative difficulties	Giant hydroceles and hernias that had stayed for over 10 years were a challenge, with no intensive care unit (ICU) facilities	Triage and referral to better facilitated centers Allocating “difficulty” cases to the experienced surgeons Priority was given to children, the elderly, and those whose conditions greatly affecting the quality of life Mainly cases which need minimal postoperative nursing care were done

Others	Only a handful presented for preoperative screening Due to limited working space, privacy could not be observed all the time Inadequate linen	Encouraging preoperative screening in future camps Procuring tents as a way of availing more working space Getting more linen

## Abbreviations

ASOU: Association of Surgeons of Uganda
GMC: Gluteal muscle contracture
HRH: Human Resources for Health.

## Additional Points

The economic and social implications of unrepaired hernia need to be investigated. For future camps, postoperative morbidity data should be collected and the total number of screened patients. Mostly importantly we consider surgical camps as an integral part of surgical service delivery by hospitals, medical schools, and Ministries of Health.
